# The Influence of Artificial Intelligence on Visual Elements of Web Page Design under Machine Vision

**DOI:** 10.1155/2022/4328400

**Published:** 2022-05-31

**Authors:** Ping Wang

**Affiliations:** School of Art Design, Shandong Youth University of Political Science, Jinan 250100, Shandong, China

## Abstract

As the presentation form of the web, web pages contain a lot of important information in web design. As many information carriers, such as sound, graphics, and text, web pages have been integrated and have played a good role in visual transmission. It inherits many features of traditional graphic design, it can be said that it extends graphic design, and web design is richer in visual performance. At present, the research on web design in China is mainly background research. There is little research on the front-end aspects of web pages, especially in the aspect of plane vision. The lack of research in this area limits the development of web design. This paper introduces the relevant situation of web design and then analyzes the relevant elements in web design, such as visual elements, and their innovative applications. In addition, it focuses on the presentation form of visual elements in web design and summarizes the development trend of visual elements in contemporary web interface design. It proposes the strategy of innovation and application of visual elements in web design. The author hopes that, through the research of this paper, it will lay a theoretical foundation for the development of web design in the future. At the same time, this paper uses the NCMF algorithm in artificial intelligence to identify the visual elements of web pages, thereby improving the efficiency of web page design. Experiments show that the Chaos accuracy rate of the NCMF algorithm in the CETD dataset is 73.3482%.

## 1. Introduction

The Internet has become an indispensable communication channel for people, and it is also an important way of information flow. Since the development of the Internet, websites are no longer simply placing information on web pages for users to browse. Now the website needs to show its own image to the outside world and needs to attract the attention of users. From the perspective of composition, the Internet is integrated through different websites, and websites are constituted by web pages. As the facade of the entire website, the web page contains visual elements that can effectively transmit information. Therefore, in the process of designing web pages, visual elements should be taken into account. It is achieved by analyzing and clarifying the relationship between visual elements and the final utilization of their respective characteristics.

In the past study, this paper has done a lot of analysis and research on the application of visual elements in web design and summed up the general requirements and laws of web interface design of several basic elements. It also focuses on the presentation of visual elements in a large sample of web pages. This paper hopes that, through this research, web designers can expand their thinking and improve the cultural connotation when designing web pages. At the same time, it also provides a reference scheme for the web design career.

The innovation of this paper is as follows:

1. This paper expounds and analyzes the composition of visual elements in web design, that is, three elements of text, graphics, and color, and explores the law of innovative application of them in web interface design.
2. It analyzes the innovation of the above three elements, studies the optimization design of the web interface, and further explores its typical style characteristics.
3. The paper tests the average recall rate and average precision rate of various algorithms in the dataset to verify the feasibility and robustness of the algorithm in this paper.

## 2. Related Work

Many scholars have provided a lot of references for research on machine vision, artificial intelligence, web design, and visual elements.

de San Eugenio Vela proposed a preliminary, exploratory, and experimental theoretical concept. He used the current consumption of the landscape as a key symbolic and physical element of territorial representation and evocation and for the deployment of local branding strategies [1].

Li et al. proposed an image representation and matching method that can significantly improve vision-based image location estimation. The example provided by Li et al. illustrates the highly transparent mechanism of this method. These mechanisms are based on commonsense observations of visual patterns in image collections [2].

Li et al. chose the application of visual design in the construction of traditional culture as a research topic. They tried to explore how to use traditional elements in visual design to promote the development of traditional culture [3].

Yang et al. proposed a method based on deep convolutional neural network (CNN) to discover discriminative midlevel visual elements. Yang et al. proposed a part-level CNN architecture, the part-based CNN (P-CNN). It acts as a coding module in the part-based representation model [4, 5].

Jankowski developed and tested a method. This approach can gradually find the optimal balance between user experience and visual intensity of website elements to maximize user conversion rates while minimizing adverse effects [6].

Under the premise of background, Jiang used eight matching methods of logo and product image elements as independent variables. He controlled the size, color, and content complexity of online advertisements through a single-factor experimental design and studied the optimal visual search rules for logo elements in online advertising formats [7].

Lee et al. proposed a new technique for classifying photographic composition rules for outdoor scenes and detecting main geometric elements called compositional elements for each composition category [8].

Saberioon et al. described the latest technologies and the applicability of different optical sensors in fish farming management, as well as the assessment, measurement, and prediction of fish product quality [9].

Shimizu et al. described an exploratory lighting design tool based on feedback from professional designers. The system extracts abstract visual objects from reference images and applies them to target regions of the stage. Shimizu et al.'s system can rapidly generate plausible design candidates embodying visual goals through Gibbs sampling methods and present them as a design library for rapid exploration and iterative refinement. Shimizu et al. demonstrated that the resulting system allows lighting designers of all skill levels to quickly create and communicate complex designs, even for scenes containing many colors [10].

Manhas discussed various traditional software and web development models commonly used in developing web applications and compared their applicability and effectiveness to develop successful web-based applications. Various reasons that differentiate traditional software development from web systems development are also identified. Finally, he proposed an initial framework that meets the needs of a specific web development process model [11, 12].

Nogueira et al. conducted an empirical test to investigate the impact of responsive design on the emotions of blind users during web interactions. In the case of responsive web design, the average number of negative emotional reactions by blind users is higher than in the case of nonresponsive web design [13].

Rusli and Luscher evaluated the ability of machine vision cameras and software to identify fasteners for assembly verification. This will enable current assembly verification systems to correlate torque verification to specific fasteners [14].

The data from these studies are not comprehensive, and the results of the studies are open to question. Therefore, it cannot be recognized by the public and thus cannot be popularized and applied.

## 3. Methods

Artificial intelligence is a branch of computer science. It seeks to understand the nature of intelligence and produce a new type of intelligent machine that responds in a similar way to human intelligence. Research in this area includes robotics, language recognition, image recognition, natural language processing, and expert systems.

The research in this paper used the methods of literature analysis, questionnaires, and interviews. In the process of conducting research, research methods are applied in combination according to the situation [15, 16].

It collects relevant literature materials, analyzes the research status and development forms related to the subject content, and uses the analysis to find a theoretical basis. It then summarizes the obtained content, which will provide a theoretical basis for subsequent research [17].

It uses the Internet to collect design examples of the website interface. In the theoretical aspect, the visual design of these examples is analyzed, and the design elements of these interfaces and the style of the interface are found through the analysis. Doing exploratory research on website style choices will make the research more actionable.

**Figure 1:**
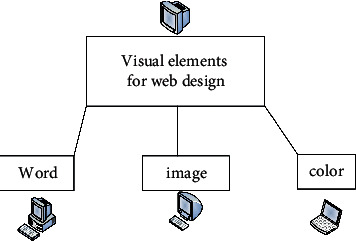
Types of visual elements in text web design.

It integrates the knowledge content and theories of related disciplines and conducts verification experiments on the subject of this research in many aspects. When designing the interface, the text needs to have prominent visual communication elements. The text in the web interface can express different levels of meaning through combination arrangement and image processing. The text design in the web interface is now the most basic expression method in information dissemination. The focus of this type of text design is the processing of font shapes and tones, and the basis is the visual expression of text styling elements. This design has now become a form of artistic expression. It can have an impact on people's emotions and perceptions through design. Web design uses this method to establish a website image and attract users to browse. The types of visual elements in web design are shown in Figure 1.

In the web interface, designers can transform ideas into forms through image design on the page and use these images to complete information transmission. Excellent page design can show the design level of the website, as well as the characteristics of the website. And it can make the information spread more vivid and three-dimensional through image design. Most designers are skilled at working with images. They believe that the communication characteristics of images are too much better than words. Others believe that the visual impact brought by images is 85% higher than the visual impact brought by text. From the above analysis, it can be seen that the images in the web page content can quickly express the designer's idea. It uses images to display information, which can bring more emotional stimulation.

**Figure 2:**
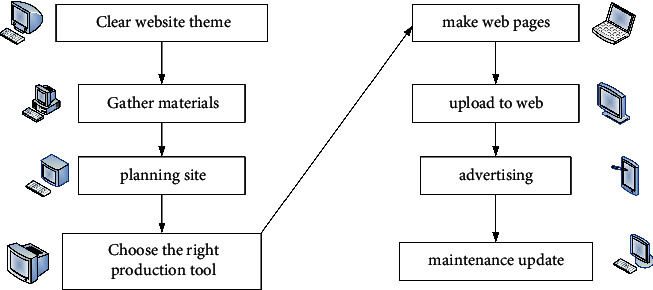
The steps of web design.

The current network technology is constantly being updated, and the related equipment is also constantly developing. Web designers now turn the graphical interface into the interface body. The text currently assumes an auxiliary role. Some designers even turn the text design into an image component so that the entire interface design has become an image design. They use such means to display their unique artistic style and enhance their visual impact. The steps of web design are shown in Figure 2.

When visual information is transmitted, color is the main body that drives emotions. Excellent color matching can attract the light of users and bring vitality to the website. The rational use of color can not only make web content more visually beautiful but also convey emotions, which is very important in the entire design process.

The color configuration in the web page will directly affect the final design effect of the web page. The colors of the various parts of the page need to match each other. How to match the color to highlight the theme of the web page is a problem that designers have always had a headache with. For an excellent web page color matching, it is necessary to first figure out what the main idea of the website is and then develop the color matching around this main body, which can increase the number of page views of the website. If it wants to achieve this effect, it needs to follow the following points:

 
*Brightness of Color*. When designing a web page, it needs to use color to make the subject more vivid so that people can notice it at first glance.
 
*The Uniqueness of Color*. The vitality of design lies in innovation. Blindly imitating will make the design lose its life, and the innovative color configuration can impress the users who have browsed it.

At present, the problems existing in the application of visual elements in web design are as follows:

The stacking of visual elements and the lack of information content.
When designing web pages, it is necessary not only to arrange the plane visual elements reasonably but also to arrange the unique elements reasonably. When designing some web pages, visual elements are randomly stacked without planning. This makes the web page lose its beauty. Elements such as animation, text, and color blocks have no design logic at all, making users easily bored. When designing web pages, new ideas must be matched with the content. Both are more important and are indispensable. Web pages need to be aesthetically pleasing, which increases page views. However, if there is only beauty and no content, it will make the web page tasteless and unable to retain users for a long time.
Interference from commercial interests.
Because the commercial economy now dominates the market, this leads to the realization of websites for profit. In order to insert advertisements into web pages, some designers use technical means to make users click on advertisements continuously in order to allow advertisements to enter the realization of browsers. This is the case on the homepage of some websites in China, and there are advertisement pictures on the page. These images partially occlude the content of the page, which is what the designers did on purpose.
One-size-fits-all imitation losing independent creativity.
Chinese web design very lacks innovation. Due to the influence of market reasons, Chinese web designers constantly refer to other countries' web design style and content, and it is very convenient to imitate other people's designs. This resulted in a lot of web pages that were very similar and completely uninspired.
Interface friendliness in dynamic interactive web design.
The difference between web design and other designs is that web design is interactive. When the interface transmits information, the effect obtained will be affected by the method. If the method is not innovative, it will cause the user to experience aesthetic fatigue, and the user's visual sensitivity will be reduced. The user's psychology and interest points are the directions that designers focus on, and the design direction of the page is formulated according to the user's psychological changes. If it has the characteristics of artistic functions and other aspects and then integrates the creativity with high recognition, it can design excellent web pages. The most common problems are as follows:
    The structural framework is unreasonable. Some websites display a lot of content on the page, which will lead to the listing of information and the lack of reasonable layout of the page. It is not easy for users to enter the page to find the desired topic. Pages need to follow short and direct principles when designing layouts. The page should not be designed too complicated.
    Web page delay: the most important thing users care about when browsing web pages is the refresh speed. If the page refresh rate is too slow when users browse, it will make users feel bored.
    The page operation is complicated. Some websites need to load plugins when registering. This increases the user's psychological pressure. If the user interface is too complex and the user cannot understand these operations in a short time, it will lead to user churn.
Visual embarrassment caused by technical factors.
Most designers have the same problem in the design process, with flaws in the handling of details, such as gradients and highlights that are too harsh, shadow distances, and opacity too high. The main reason for this is that the designer is not fully engaged. Web designers need to keep in mind that the most critical part of web design is the handling of details. If the technical ability is not enough, the creative realization cannot be completed. Therefore, it is necessary to use practice to continuously improve the technique in this area so as to avoid visual defects.

**Figure 3:**
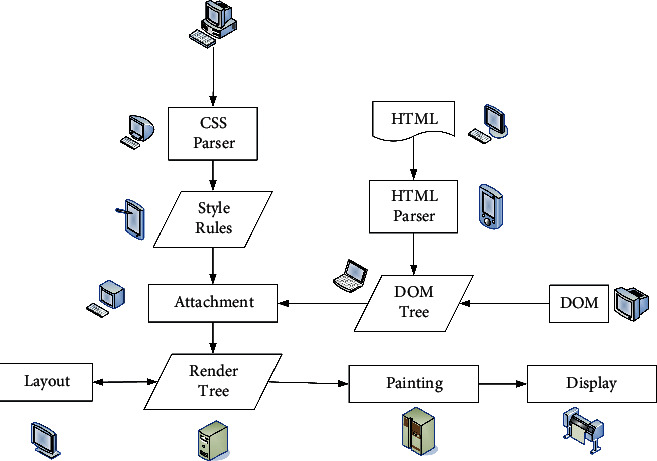
Web page rendering process.

**Figure 4:**
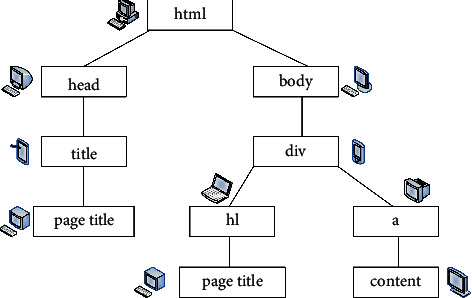
The DOM tree corresponding to the web page code snippet.

The innovative use strategies of visual elements in web design are as follows:

  Innovative use of text elements.
  When designing an interface, text is the most basic element of web design. Designers should use the text according to their own needs. Text has a higher accuracy of delivery. When designing a font, it needs to consider the overall layout of the interface and the situation of the user. Usually, the default mode is selected when the font is used. The use of special fonts may not necessarily achieve the expected results, and such fonts have very large limitations, but the text download speed is very fast.
  Text needs to be simple and easy to understand, especially when designing a page. When color matching, the text should be accurately and reasonably configured with various parameters. The distance between text and text needs reasonable design. When configuring fonts, designers need to be fully committed, fully consider the details, and not make low-level mistakes, which will make the page design very unprofessional for users to see. Font design can perfectly highlight the main body of the design, and it is also an important part of realizing the idea. When designing, it is necessary to highlight the style and bring in new ideas. It is necessary to repeatedly consider and add certain graphic texts. In short, the design considers details and highlights individual characteristics.
  Using the color of text in web design can play an eye-catching role and quickly attract the attention of web browsers. And certain color matching can also reflect the theme of the page. The use of text emotion on web pages is to use text size, arrangement, or spatial hierarchy to realize web page design. For example, in a free and leisurely web design theme, loose and random text arrangement is used, and a neat structure can reflect the characteristics of rationality and prudence.
  Innovative use of graphic elements.
  Various drawing software such as Photoshop, Painter, and CorelDRAW provide richer content for graphic design. Graphics images come into web design in the early days. It is more visual and intuitive than text in terms of information transmission and also plays a role in beautifying the page. In the traditional design, the parameter setting requirements for the image are relatively high. However, the requirements for resolution of current network pictures are not very high. Since the network pictures are basically only displayed on the computer, the pictures will be affected by the resolution of the display itself. Web page images basically rely on broadband to spread. The smaller the file size in the corresponding range is, the better it is.
  Graphic design generally includes six modeling techniques: generalization, symbolization, abstraction, figuration, comics, and decoration. Generally, the generalization is to describe the object by the most direct and simple method, and the generalization affects the recognition accuracy of the web browser. Figurative can vividly express the natural form, which is real, clear, and convincing. The use of abstraction has the characteristics of concise language, which gives people a sense of profound meaning visually, has a large space for association, and has a strong sense of design. In the process of designing web pages, the above six graphic design methods are often used in combination, infiltrating and influencing each other. The innovative use of graphic elements is to use the representation of graphs to produce very distinctive and artistic web pages. The rendering process of the web page is shown in Figure 3.
  Many of the images used in web design are real photos, which can be expressed directly and concretely. It uses postprocessing to transform the original image to form a picture with good artistic effect and in line with the theme of web page design. Therefore, the design of images on web pages can effectively express creativity and ideas.
  Innovative use of color elements.
  Color is the main visual element in page design. If the use of color is reasonable, it can greatly improve the beauty of the page. When the web page is designed, it is launched on the network platform, so it needs to follow international standards. This is also often referred to as a web-safe color. The user's emotions can be mobilized through color rendering. There are basically no restrictions on the use of colors on web pages, but it is not possible to use colors at will. Under normal circumstances, web designers need to determine the primary and secondary colors according to the theme.
  The use of color in web design is chosen according to different themes and preferences. It can choose a single color, two colors, or the same color. First of all, the emergence of the Semantic Web provides possible preconditions and new technical support for the requirement of network intelligence. The Semantic Web is simply the ability of the web to recognize and understand keywords defined by people. It then searches according to the definition of the word or information related to the word, forming a relatively simple “dialogue communication” between people and networks. Although it enables the intelligentization of the network to be realized, it still cannot meet the more and more ideal pursuit of users for the visual effects of the web interface. Therefore, both CSS and HTML have developed and updated new versions-CSS3 and HTML5 in accordance with this demand trend. Their appearance provides new and better conditions for the presentation of various visual elements in web design and the presentation of overall visual effects. Now the CSS3 + HTML5 web design method has been accepted by most designers and has gradually been widely used. Compared with the previous HTML, HTML5 supports the presentation of more visual elements on web pages, and all have very clear and smooth visual effects. The performance and compatibility are more stable than the previous ones, and the adaptability is better in different versions of browsers. This series of changes in HTML and CSS has brought a strong impact to Flash, which once occupied the mainstream of web design. However, the maturity of any emerging technology requires a certain amount of development time, and there is still a certain gap between HTML5 and Flash. Designers nowadays are more using HTML5 combined with Flash. They each draw on their strengths. This makes the web page visually richer, lighter in size, and easier to load. Among them, CSS3, which is closely related to web design, is becoming more and more applicable. The visual effects of some visual elements are set with CSS3, which is even easier to operate, more convenient and faster, and better than Photoshop. Because of the comprehensive opening and support of emerging technologies such as CSS3 and HTML5, web design between 2010 and 2016 has formed a new fashion trend. Individual visual elements have also changed a lot in this new trend. The DOM tree corresponding to the web page code fragment is shown in Figure 4.

In terms of layout, special layouts, multicolumn layouts, and single-page layouts have emerged.

The biggest difference between out-of-format layouts and traditional layouts is that they break the rules. The page division is no longer neat squares, and there are special-shaped divisions. This gives the original two-dimensional interface and a three-dimensional visual effect. This type of layout emphasizes individuality and innovation, which can attract more attention visually and make people memorable.

Multicolumn layout is supported by CSS3 Grid layout. The advantage of the multicolumn layout is that it can display different pieces of content on one page. However, users will not confuse the content of each column and can also form a logical relationship between multiple columns, complementing and extending each other. This allows users to learn as much as possible about the information the web page is going to display on a page. Multicolumn layouts have also appeared before. But with the growth of CSS, page segmentation is no longer rigid. The block surface is no longer a simple and rude division of large blocks but becomes small and agile, and the arrangement is also flexible. However, the multicolumn layout tests the designer's typography ability. It needs to have a clear structure, clear priorities, no clutter, and proper white space. This creates a staggered visual effect that increases the appreciation of the web page.

The one-page layout is characterized by the fact that part of the page does not change. According to the mouse click on the navigation label or button, or even the text link, the whole page is no longer jumped. It produces partial, modular swipe or page jump changes directly on the page. In this way, the layout content is listed and hidden and is revealed layer by layer as the click. This fresh, animated, and highly interactive visual effect is achieved thanks to the combination of HTML5 and JavaScript. Their powerful in-app web pages have made one-page layouts one of the typographical trends among the new trends in web design.

Similar to the one-page layout is the thumbnail layout. This layout design relies on browse preview technology. It hovers over the link with the mouse but does not click, and after a few seconds, there will be a preview image and a brief text content floating on the main page. The advantage is that more specific content is displayed, but it does not jump on the page, which is convenient for users to find the information they need as soon as possible.

On color elements, the function of HSL colors in CSS3 provides support conditions for the realization of the above-mentioned effects of colors. This makes simple color schemes popular. Designers have noticed that a solid-color background is easier to attract users' attention, and it is more intuitive and concise in expressing content. Because colors can bring different emotional effects on people, designers will consider many factors such as the nature of the web page, the purpose of the web page, and the audience when choosing the main color of the web page. But the general consensus is that the number of colors cannot be too much. One can be used as the main color, and 2 to 3 colors can be used as auxiliary colors. It adjusts the transparency of the color, and it is also one of the more brilliant visual effects that color elements can bring to users in web design.

The simple red, yellow, and blue colors are matched, and the main content is placed on the yellow color block that occupies a large visual area. Its content is clear and clear, and the overall visual effect is concise and generous. The bright light blue area becomes the first focal point of the eye. The large-scale layout not only highlights the white main body in the center of the web page but also sets off the three white functional blocks below. The small icons on the three functional areas all have the same light blue color, which makes the interface as a whole echo each other and is exquisite and interesting.

In terms of graphic elements, first of all, photos are an important part of graphic elements. With the powerful functions of Photoshop and the increasingly sophisticated photography technology, it has been loved by designers. Large background images have become the new trend. The use of pictures to narrate events has become the new form of content expression. Because of the realism of photos, it can bring great visual impact to users and increase the attractiveness of web interface. The Canvas in HTML5 and the Background series tags in CSS3 are the technical conditions for realizing large background images.

Coordination is the most important part of the design of the large background image web interface. The color and content of the background image cannot be arbitrarily matched, drowning the main content of the page. The pictures should be soft and slightly transparent, should not affect the text display of the website, and should be easy to read. Secondly, graphic design software such as Photoshop and AI have achieved further harmony and coordination due to the increased functions and powerful technologies of HTML5 and CSS3. The designed graphic patterns with various styles can be well displayed in the web page, so that the visual effect styles brought by the design pictures are diversified, and the selection space of the visual effect of the web page interface is enriched.

Finally, an important design part of the graphic element is the icon. Icons are used as a guide and identification on web pages, and a high degree of generalization and simplification of symbols has become hard standards under the new fashion trend. This requires designers to have a thorough understanding of the functionality a website can provide when creating favicons. The development of icons has several stages, such as “conversational navigation,” flat design, and the joining of Material Design.

Limited by the indication function of the navigation bar, it is obviously difficult to summarize the powerful functions in one word. The designer chose to explain it in one word, and this way of designing it is like trying to have a dialogue with the user, so it is called “conversational” navigation.

Flat design is a highly refined graphic symbolization, removing redundant decorative effects. It allows the information represented by the graphic itself to stand out as the core. And the design emphasizes abstraction and simplification. Not only has the popularity of flat design appeared on the icon, but also all graphic elements of the entire page have been affected by it.

Material Design is actually a new visual design effect in the development of flat design. Compared with flat design, which blindly emphasizes the refinement and abstraction of symbols, it likes the addition of colorful colors. This allows for the presence of texture, texture, light, and shadow, like a certain dimensionality and quality. It is now also one of the styles that influence the design of the overall web interface graphic elements.

As the main way of narrative expression in web interface, font elements have always been valued by designers. However, due to the limitations of technical conditions and reading rules, the style of the font has not changed much. Until the advent of tags such as text-shadow and text-overflow in CSS3, designers began to present the visual effect of fonts as a graphic pattern in the interface.

In the typesetting of a large amount of text, it is no longer rigidly bound to the traditional four-square pattern. With the vertical and horizontal development of web page reading, the irregular layout of printed materials such as fashion magazines and newspapers has been sought after and has become a new trend of text content arrangement.

HTML5's powerful multimedia support function enables video and audio to appear in the web interface more smoothly and clearly. Because the audio and video of the video are synchronized, it is more realistic and visually impactful than the picture. With this advantage, video, as a new narrative method, is gradually replacing the narrative method of browsing multiple large pictures. Audio appears in the opposite way to video; except for dedicated music websites, music is no longer a function, but a semihidden appearance. Styles continue to be so simple that users can even ignore their existence. It is more just to make the background music flow naturally and harmoniously integrate with the overall visual effect of the page.

Between 2010 and 2016, a new way of browsing the web was born, that is, the mobile web. With the popularization of mobile terminals such as mobile phones and iPads, it has entered people's field of vision. But also because of the rapid development of this quicker, easier, and portable browsing mode, new requirements have been put forward for the design of the web interface. Visual elements are also affected and changed, such as typographic layouts, adding responsive layouts. It appears to solve the problem of dislocation and deformation of the originally designed web page interface due to different page sizes of different mobile terminals.

Although Media Query in CSS3 can flexibly change the interface size of web pages, there are still some shortcomings such as low efficiency and unstable loading. It needs further improvement of technology, but responsive layout will undoubtedly become the mainstream of future web layout. The development of responsive layout has led to another layout style. It integrates graphic elements such as icons with functional modules, which is called modular layout. This layout form in CSS has certain content display shortcomings. Because it mainly distinguishes the primary and secondary levels by the size of each module, it is easy to create a situation of equal size, blur the boundaries, and cause trouble for users. But it perfectly solves the problem of adjusting the layout with the page size. Because each module can change its size to fit the space as the screen size changes, this division method is obviously more suitable for mobile web pages.

The changes in graphic elements, color elements, and font elements in mobile web pages are concentrated in the design of icons. Because the interaction between mobile web pages and users is no longer a mouse click, a design change brought about by the technological progress of the touch screen can sense the force of the screen. It is whether the icon can satisfy the visual effect of fingertip touch so that users feel that buttons and links are indeed triggered. Therefore, the icons on the mobile web page emphasize the visual texture more than the icons on the web page.

The comprehensive and open development of contemporary Internet technology has made the visual effects of web design present a situation where a hundred flowers are blooming. Different styles of web interface give users more choices for a beautiful and smooth browsing experience. But it also means more updated requirements for visual elements in web design. How will the visual elements change in the future and which Internet technologies will emerge bring new methods and technical support to web design. These kinds of curiosity will inspire web designers in modern and contemporary times to grow up with the visual elements of web pages and Internet technology.

## 4. Simulation Experiment Analysis and Results

This assumes that given a training sample set, *χ*={(*μ*_1_, *ν*_1_), (*μ*_2_, *ν*_2_),…, (*μ*_*ξ*_, *ν*_*ξ*_)}, where *μ*_*σ*_ is the feature vector representation of a training sample. *ν*_*σ*_ indicates the category it belongs to, and its value can be +1 or −1, indicating two different types given, respectively.

(1) $$\begin{array}{c} \delta •\mu +\alpha =0. \end{array}$$

**Figure 5:**
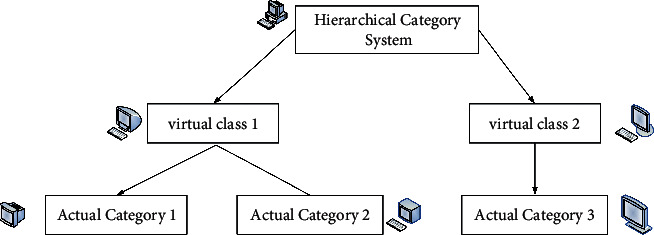
Virtual category tree structure.

The virtual category tree structure is shown in Figure 5.

For sample *μ*_*σ*_ in the training sample set, it satisfies the following relationship:

(2) $$\begin{array}{c} \delta •\mu_{\sigma }\geq +1,\;\nu_{\sigma }=+1,\\ \delta •\mu_{\sigma }\leq +1,\;\nu_{\sigma }=-1. \end{array}$$

That is,

(3) $$\begin{array}{c} \nu_{\sigma }\left(\delta •\mu_{\sigma }+\alpha \right)\geq 1. \end{array}$$

The optimal decision function is as follows:

(4) $$\begin{array}{c} \gamma \left(\mu \right)=\text{sign}\left(\delta •\mu_{\sigma }+\alpha \right). \end{array}$$

The distance from any sample ${\overset{-}{\mu }}_{\sigma }$ in the training sample set to the optimal classification surface is as follows:

(5) $$\begin{array}{c} \varepsilon =\frac{\left\|\delta •{\overset{-}{\mu }}_{\sigma }+\alpha \right\|}{\left\|\delta \right\|},\\ \varphi =\frac{2}{{\left\|\delta \right\|}^{2}}=\frac{2}{\delta •\delta^{T}}. \end{array}$$

(6) $$\begin{array}{c} \min\frac{1}{2}{\left\|\delta \right\|}^{2},\\ s.t.\nu_{\sigma }\left(\delta •\mu_{\sigma }+\alpha \right)\geq 1\left(\sigma =1,2,\ldots ,\xi \right). \end{array}$$

It introduces a nonnegative slack variable *η*=(*η*_1_, *η*_2_,…, *η*_*ξ*_), which transforms formula (3) into

(7) $$\begin{array}{c} \nu_{\sigma }\left(\delta •\mu_{\sigma }+\alpha \right)+\eta_{\sigma }\geq 1. \end{array}$$

It introduces a penalty constant of *κ*, which transforms formula (6), into

(8) $$\begin{array}{c} \underset{\delta ,\alpha ,\eta_{\sigma }}{\min}\frac{1}{2}\delta^{T}•\delta +\kappa \sum_{\sigma =1}^{\xi }\eta_{\sigma },\\ s.t.\nu_{\sigma }\left(\delta •\mu_{\sigma }+\alpha \right)\geq 1-\eta_{\sigma }\eta_{\sigma }\geq 0,\;\sigma =1,2,\ldots ,\xi ,\\ \max\varphi \left(\beta \right)=\sum_{\sigma =1}^{\xi }\beta_{\sigma }-\frac{1}{2}\sum_{\sigma =1}^{\xi }\sum_{\varsigma =1}^{\xi }\nu_{\sigma }\nu_{\varsigma }\beta_{\sigma }\beta_{\varsigma }\psi \left(\mu_{\sigma }•\mu_{\varsigma }\right),\\ s.t.\sum_{\sigma =1}^{\xi }\nu_{\sigma }\beta_{\sigma }=0,0\leq \beta_{\sigma }\leq \kappa ,\;\sigma =1,2,\ldots ,\xi , \end{array}$$

where *β*=(*β*_1_, *β*_2_,…, *β*_*ξ*_) represents the Lagrange multiplier.

The weight of the optimal classification surface is as follows:

(9) $$\begin{array}{c} \rho =\sum_{\mu_{\sigma }\in \varpi }\beta_{\sigma }\nu_{\sigma }\mu_{\sigma }, \end{array}$$

where *ρ* represents the support vector set.

The optimal classification function is as follows:

(10) $$\begin{array}{c} \gamma \left(\mu \right)=\text{sign}\left(\sum_{\mu_{\sigma }\in \varpi }\beta_{\sigma }\nu_{\sigma }\mu_{\sigma }+\alpha \right). \end{array}$$

Sample set is as follows:

(11) $$\begin{array}{c} \tau =\left\{\tau_{\sigma }|\tau_{\sigma }\in {Ζ}_{\sigma }\right\}, \end{array}$$

where *Z* is a category.

Mapping is as follows:

(12) $$\begin{array}{c} M:\tau *Z⟶\left\{I,K\right\}, \end{array}$$

where *I* and *K* represent function values.

Approximate function is as follows:

(13) $$\begin{array}{c} \overset{\wedge }{M}:\tau *Z⟶\left\{I,K\right\}. \end{array}$$

New unknown objective function is as follows:

(14) $$\begin{array}{c} M^{\prime }\left(\tau ,Z_{\sigma }\right)=I⟹M^{\prime }\left(\tau ,Z_{\varsigma }\right)=I. \end{array}$$

**Figure 6:**
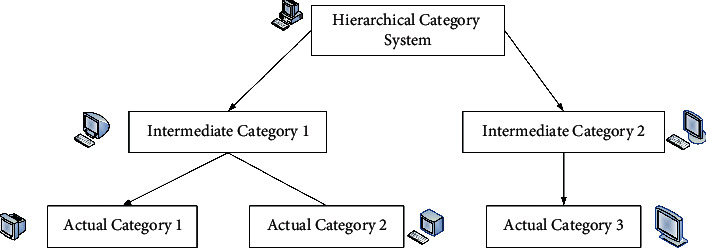
Category tree structure.

And the relationship is satisfied between the two categories:

(15) $$\begin{array}{c} Z_{\varsigma }⟶Z_{\sigma },Z_{\sigma },Z_{\varsigma }\in T, \end{array}$$

where *T* is the hierarchy of categories. The category tree structure is shown in Figure 6.

Vector distance calculation is as follows:

(16) $$\begin{array}{c} \zeta \left(\mu ,\mu_{\sigma }\right)=\sqrt{\vartheta \left(\mu ,\mu \right)-2\vartheta \left(\mu ,\mu_{\sigma }\right)+\left(\mu_{\sigma },\mu_{\sigma }\right)}. \end{array}$$

The ratio of the number of support vectors contained in each of the positive and negative categories is as follows:

(17) $$\begin{array}{c} ο=\frac{\text{Count}\left(\mu_{\sigma }^{+}\right)}{\text{Count}\left(\mu_{\sigma }^{-}\right)}. \end{array}$$

The experimental dataset in this paper is to use web crawler to crawl 6000 web pages and automatically extract features and label categories for CSS DOM nodes in each web page through the program to obtain labeled feature data. There are a total of 1,230,756 labeled feature data, and the dataset is divided and tested by 5-fold cross-validation.

In this paper, five commonly used machine learning classification algorithms are selected in the comparison algorithm, namely, LR, GBDT, XGBoost, random forest, and deep neural network. This paper first compares these five machine learning classification algorithms on complete experimental data to compare the effects of each machine learning classification algorithm on the dataset. Then, this paper uses the XGBoost classification algorithm to compare the classification effects of only using text features, only using visual features, and using both text features and visual features. It thus compares the influence of feature selection on classification results.

**Figure 7:**
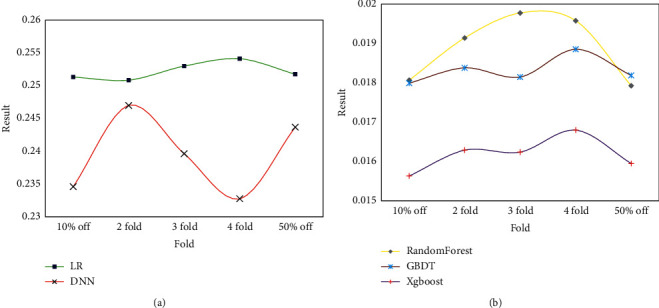
Comparison results of different classification algorithms for CSS DOM node classification.

The XGBoost classification algorithm uses XGBoost, LR, and GBDT in Python. The random forest classification algorithm uses a good algorithm implemented in the sklearn package in Python. The deep neural network classification algorithm uses a network built with the TensorFlow framework. The comparison results of the classification effects of different classification algorithms on CSS DOM nodes are shown in Figure 7.

Figure 7 shows that the accuracies of the three tree classification models are relatively close, and they are all better than the LR model and the DNN model. Analysis of the reason found that this is because the experimental sample is extremely dense; that is, the number of features of the sample is small, and the amount of data of the sample is large. Such data will have better results using a tree model. The effect of using LR, SVM, neural network, and other models will be poor. Therefore, the tree model will have a better classification effect for dense samples, while models such as LR, SVM, and neural network are more suitable for sparse samples. It can also be seen from Figure 7 that the accuracy of the tree model is very high, and the average logloss is lower than 0.02. This shows that the features selected in this paper have a good degree of discrimination for the experimental samples and can distinguish the text content and the noise content on the web page very well.

**Figure 8:**
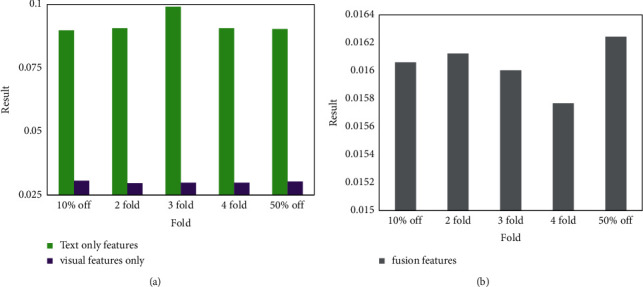
The impact of feature selection on CSS DOM node classification results.

This section will use the XGBoost classification algorithm to compare the classification effects of using only text features, using only visual features, and using both text features and visual features. Because the algorithm in this section extracts and classifies the CSS DOM nodes of the web page, the web page structure features are implied in the feature data, and therefore the web page structure features are not discussed in this experiment. The experimental results are shown in Figure 8.

Figure 8 shows that the logloss of classification results using only textual features or only visual features is lower. This shows that text features or visual features can have a good degree of discrimination for sample data. At the same time, the results using only visual features are better than those using only text features, which shows that visual features are more effective than text features in the CSS DOM node classification task. However, the classification results using only textual features or only visual features are worse than those using both textual and visual features, which indicates that feature fusion is beneficial for CSS DOM node classification.

The experimental results of the three comparison algorithms on the CETD dataset are shown in Tables 1–3.

Tables 1–3 show that the accuracy rate of the BBC of the newspaper algorithm in the CETD dataset is 84.4255%, and the recall rate is 70.7658%. At the same time, both the precision rate and the recall rate of the three algorithms have reached the general requirements of web page extraction.

**Figure 9:**
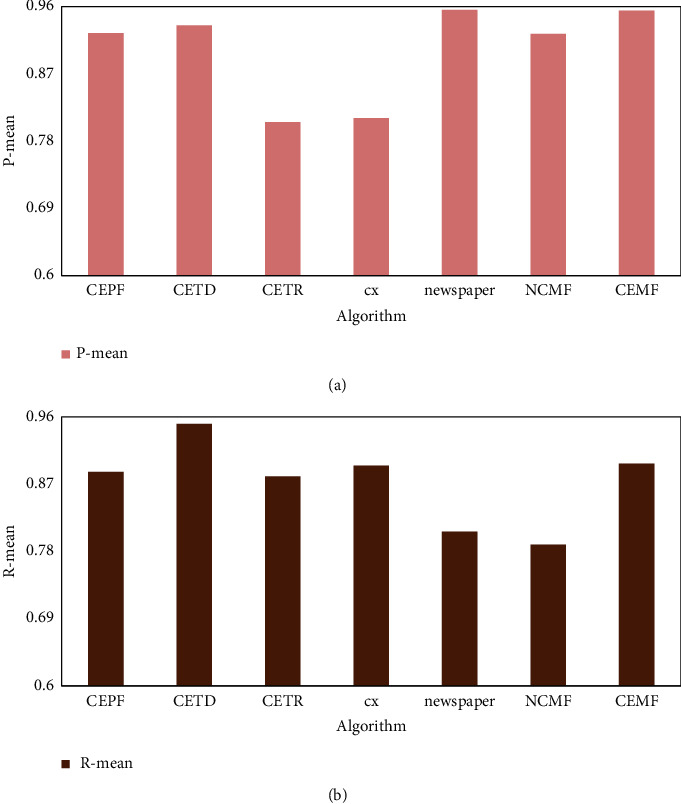
Average precision and average recall of each algorithm on the CETD dataset.

The average precision and average recall of each algorithm on the CETD dataset are shown in Figure 9.

Figure 9 shows that the average precision score of the CEMF algorithm on the CETD dataset is only lower than that of the newspaper algorithm, and the average recall rate is only lower than that of the CETD algorithm, indicating that the CEMF algorithm has a good extraction effect on the content of web pages.

The experimental results of the three comparison algorithms on the CETR dataset are shown in Tables 4 and 5.

Tables 4 and 5 show that the Freep accuracy of the newspaper algorithm in the CETR dataset is 77.8322%, and the recall rate is 94.1287%. The efficiency of the other three comparison algorithms in the CETR dataset can already meet the general needs.

**Figure 10:**
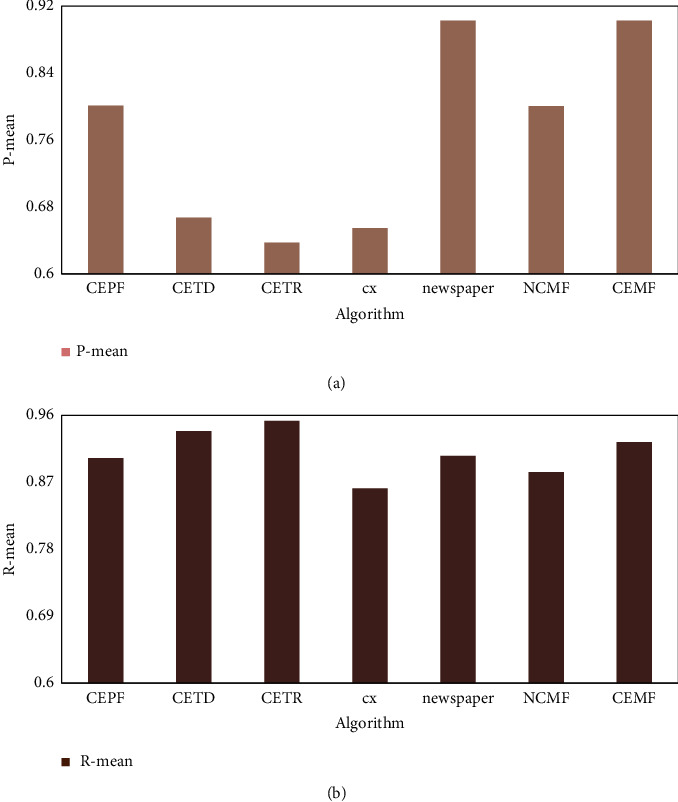
Average precision and average recall of each algorithm on the CETR dataset.

The average precision and average recall of each algorithm on the CETR dataset are shown in Figure 10.

Figure 10 shows that the CEMF algorithm also has the highest average precision on the CETR dataset, and the average recall is lower than the CETR and CETD algorithms. This shows that the CEMF algorithm still has a good extraction effect on the CETR dataset. Although the CETR algorithm and CETD algorithm can still maintain a high recall rate on the CETR dataset, the accuracy rate is very low.

## 5. Discussion

The web interface incorporates various elements such as color, text, and graphics. It realizes information transmission through the combination and arrangement of responses and undertakes the medium of people's communication. Interface design is the integration of technology and visual elements. It interprets functions and forms through external performance and uses comprehensive considerations to achieve users' psychological expectations. Web design needs to follow people's visual laws, people-oriented but also need to bring its own style. When designing web pages, form and content need to be perfectly integrated and unified with each other.

Web design has played an irreplaceable role in the current social form. It extends and develops art forms through the fusion of technical and visual elements. With the advancement and popularization of network technology, it is extremely common in life. Of course, there are also many areas that need to be improved. This requires us to continuously analyze and study the problems that arise and generate new thinking and understanding.

The CEMF algorithm used in this paper not only passed the test requirements but also played an important role in text extraction in web page design.

## 6. Conclusion

In order to solve the problem of the complexity of visual elements in web page design, this paper uses artificial intelligence algorithms to collect and analyze the visual elements of existing web page design. In addition to the types of existing web page design visual elements, it can be divided into three types: text, graphics, and colors. At the same time, it analyzes the application of these elements in web design, including article typesetting, graphic design, and color matching, which provides a reference scheme for the editing of visual elements of web design and provides a reference for the many aspects involved in artificial intelligence. The experiment was carried out on various algorithms, and an algorithm suitable for the extraction of visual elements of web page design was found. However, this study did not practice the application of visual elements on web pages, so the effect of applying the visual elements given in this paper to web design remains to be studied. In addition, many of the contents of this paper are not in line with the times, and many of the materials are relatively outdated, so it is recommended to update the content of the paper.

## Figures and Tables

**Table 1:** Extracted *F* − 1 values of the algorithm on the CETD dataset.

Algorithm	Newspaper	NCMF	CEMF
BBC	0.748156	0.736403	0.771433
Chaos	0.710572	0.687439	0.824437
Wiki	0.667342	0.667128	0.834767

**Table 2:** The extraction accuracy of the algorithm on the CETD dataset.

Algorithm	Newspaper	NCMF	CEMF
BBC	0.844255	0.78352	0.844815
Chaos	0.78346	0.733482	0.855724
Wiki	0.876282	0.873327	0.814227

**Table 3:** Extraction recall of the algorithm on CETD dataset.

Algorithm	Newspaper	NCMF	CEMF
BBC	0.707658	0.71515	0.751678
Chaos	0.655618	0.631146	0.775234
Wiki	0.544857	0.54572	0.835123

**Table 4:** The extraction accuracy of the algorithm on the CETR dataset.

Algorithm	Newspaper	NCMF	CEMF
Freep	0.778322	0.636426	0.778241
Nypost	0.757655	0.735415	0.754613
Nytimes	0.683225	0.642663	0.676534

**Table 5:** Extraction recall of the algorithm on CETR dataset.

Algorithm	Newspaper	NCMF	CEMF
Freep	0.941287	0.817673	0.843128
Nypost	0.662443	0.662381	0.734167
Nytimes	0.721538	0.721175	0.732718

## Data Availability

The data used to support the findings of this study are available from the corresponding author upon request.
